# Cholesterol, Statins, and Alzheimer Disease

**DOI:** 10.1371/journal.pmed.0020081

**Published:** 2005-03-29

**Authors:** Alexei R Koudinov, Temirbolat T Berezov

**Affiliations:** Russian Academy of Medical SciencesMoscowRussia

After reading the excellent research article by Pedrini et al. [1] and the associated synopsis [2], one may conclude that the only pathway of statins' effect on Alzheimer disease (AD) is the regulation of amyloid precursor protein (APP) processing and amyloid-ß protein (Aß) generation. The moderation is provided in the research article's patient summary, reminding that “statins are likely to influence the risk for Alzheimer disease by several different pathways.” What are these other pathways? It is essential to note that in addition to APP processing and Aß chemistry being modulated by statins, fine tuning of cholesterol homeostasis also affects cholinergic function, ionotropic and metabotropic receptors, tau phosphorylation, neural oxidative stress reactions, and other features of neurodegeneration (reviewed in [3]). Moreover, precise regulation of neural cholesterol dynamics and supply is itself essential for synapse function, plasticity, and behaviour [3]. These data suggest that in addition to its role in sporadic AD, cholesterol homeostasis break is the unifying primary cause of neuromuscular diseases, Niemann-Pick type C disease, and Down syndrome, and explains why rare cases of familial AD (associated with mutations in APP and presenilin genes) are translated into Alzheimer's via membrane cholesterol sensitivity of APP processing by secretases and Aß generation. Also important, is the synopsis's [2] apparently outdated dividing of APP processing into “harmful” (Aß-generating) and “healthy” (non-amyloidogenic). One should be cautious in calling Aß a harmful molecule. This is because several recent studies have illuminated an essential function for amyloidogenic processing of APP and Aß in neurons [4] and synapses [5]. In this context, the reciprocal effect of Aß on cholesterol synthesis, cellular uptake, efflux, and esterification, and its relation to the experimental restoration of long-term potentiation (LTP, a synaptic plasticity measure) may represent one of the poorly comprehended physiological functions of Aß [6,7].
